# High Thermal Conductivity of Copper Matrix Composite Coatings with Highly-Aligned Graphite Nanoplatelets

**DOI:** 10.3390/ma10111226

**Published:** 2017-10-25

**Authors:** Alessandro Simoncini, Vincenzo Tagliaferri, Nadia Ucciardello

**Affiliations:** Department of Enterprise Engineering “Mario Lucertini”, University of Rome “Tor Vergata”, Via del Politecnico 1, 00133 Rome, Italy; tagliaferri@mec.uniroma2.it (V.T.); nadia.ucciardello@uniroma2.it (N.U.)

**Keywords:** graphite nanoplatelets, polarized Raman spectroscopy, anisotropy phonon thermal conductivity, metal matrix composite coating (MMCC), thermal analysis

## Abstract

Nanocomposite coatings with highly-aligned graphite nanoplatelets in a copper matrix were successfully fabricated by electrodeposition. For the first time, the disposition and thermal conductivity of the nanofiller has been evaluated. The degree of alignment and inclination of the filling materials has been quantitatively evaluated by polarized micro-Raman spectroscopy. The room temperature values of the thermal conductivity were extracted for the graphite nanoplatelets by the dependence of the Raman G-peak frequency on the laser power excitation. Temperature dependency of the G-peak shift has been also measured. Most remarkable is the global thermal conductivity of 640 ± 20 W·m^−1^·K^−1^ (+57% of copper) obtained for the composite coating by the flash method. Our experimental results are accounted for by an effective medium approximation (EMA) model that considers the influence of filler geometry, orientation, and thermal conductivity inside a copper matrix.

## 1. Introduction

Electronics have been the major growth sector in the world economy over the past two decades. To sustain this growth, modern electronics need better cooling technologies to compensate their increasing power densities, along with weight and size reductions [1]. Accordingly, low-dimensional carbon structures have revealed truly exciting features for heat removal and thermal conductivity [2,3]. In the last few years, great efforts were invested to use the excellent mechanical and thermal properties of nano-sized carbon materials, like carbon nanotubes (CNTs) [4,5], graphene [6,7] or graphite nanoplatelets (GnPs) [8], and nano-diamonds [9,10] to refine a copper matrix [11,12]. In this regard, highly-ordered pyrolytic graphite (HOPG) is undoubtedly attractive for its excellent anisotropic thermal properties, with an in-plane thermal conductivity of approximately of 2000 W·m^−1^·K^−1^ at room temperature [13,14,15,16], and low production cost. Due to the outstanding thermal properties of graphite, high-purity nano-crystals have been recently used as fillers for metal matrix composites (MMCs) to conduct heat efficiently [17,18,19,20]. However, the alignment and dispersion of carbon nanofillers inside the metal matrix play the most relevant role for the increase of heat conduction [21,22,23,24,25,26,27,28]. For MMCs, carbon nanofillers are usually difficult to disperse uniformly within the metal matrices. In traditional powder metallurgy processes, several groups showed that carbon nanoparticles have a strong tendency to agglomerate due to Van der Waals forces. Zhang et al. [29] reported that GnPs strongly agglomerated in a Cu-matrix with 0.5 vol % of GnPs; while Bartolucci et al. [30] reported that the MMC with 0.1 wt % of multi-layer graphene (MLG) in an Al-matrix had a lower strength with respect to the pure Al counterpart due to agglomeration. Nevertheless, in recent years many different processes have been developed to solve the agglomeration problem, such as molecular-level mixing [31,32], electroless plating [29], or surface modification of carbon fillers and Cu powder by polymers before mixing and sintering [33]. However, achieving a high degree of alignment in a reproducible way remains challenging. Khaleghi et al. [34] used strong magnetic fields to align copper-coated carbon nanotubes within a copper matrix. Compared to unaligned samples, they obtained a thermal conductivity enhancement along the nanotube main direction by a factor of four. Firkowska et al. [21] used the GnPs size to control the alignment and thermal conductivity in copper matrix composites, showing that the alignment is inseparably linked to the lateral size of the nanoplatelets. This relationship resulted in anisotropic thermal properties of the composites, with a thermal conductivity along the flake alignment direction up to five times higher than perpendicular to it. In this context, electrochemical processes allow the realization of metal matrix composite coatings (MMCCs) with a better control of the alignment and inclination of the nanofillers, combined with excellent dispersion. Such copper-matrix composites with highly-aligned GnPs have great potential in directed heat dissipation applications, allowing an in-plane thermal conductivity above pure copper. In this paper, we used the electrochemical method developed by Antenucci et al. [35] to synthesize thin composite coatings with highly-aligned graphite nanoparticles. For the first time, the carbon nanofillers inside the copper matrix and the entire composite structure have been entirely characterized. In this way it was possible to refine a whole series of indispensable characterizations to understand the present and future potentiality of the process. The orientation and inclination of the nanoplatelets in the copper matrix were verified by polarized Raman scattering. The room temperature thermal conductivity of 1244 ± 86 W·m^−1^·K^−1^ of the nanoparticles has been measured by the dependence of the Raman G-peak frequency on the laser power excitation and independently measured G-peak temperature coefficient. The resulting composite coating has a superior thermal conductivity of 640 ± 20 W·m^−1^·K^−1^ evaluated by the flash method, which is in excellent agreement with modeling based on the effective medium approximation (EMA). 

## 2. Experimental

### 2.1. Sample Preparation

**Figure 1:**
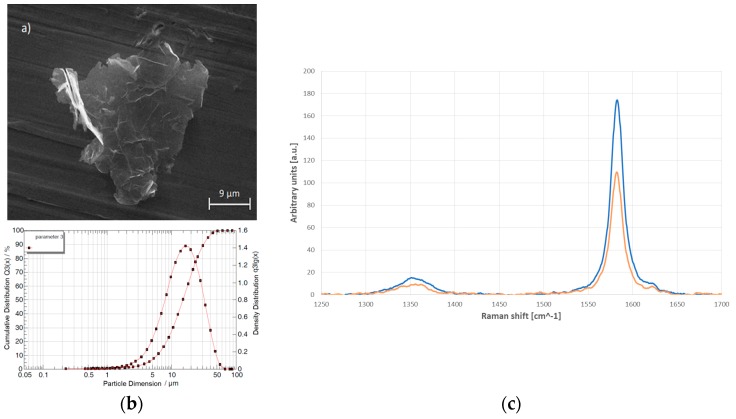
GnPs characterization: (**a**) SEM image of a single nanoplatelet of graphite; (**b**) particle size analysis of GnPs, as provided by the supplier; and (**c**) representative Raman spectra of GnPs before (blue line) and after (orange line) the electrodeposition process.

Graphite nanoplatelets obtained by exfoliation of expanded graphite, provided by NANESA s.r.l. (Arezzo, Italy), were used to realize an electrochemical metal matrix composite coating (MMCC) able to improve the thermal properties of aluminum substrates. 6082-Aluminum alloy samples have been chosen as substrates for the electrodeposition process. The surface preparation was carried out using a sandblasting machine to easily remove the oxide layer that is formed on the surface. The electrodeposition process used is composed of three distinct phases [35,36]. During the first phase, a thin copper layer is electroplated on the aluminum substrate to prepare the surface for the GnP deposition, given the high electron affinity between the two materials. During the second phase the GnPs and Cu are simultaneously electroplated. In this phase, the inclination of the nanoparticles can be adjusted by controlling the growth of copper crystal. Due to the extreme volatility of GnPs, during the third phase a thin copper layer is electroplated to trap the nanoparticles inside the coating. For the entire electrodeposition process a copper sulphate solution, with water as the solvent, has been used as an electrolytic bath. For the first and third phases, an acidic bath was used at room temperature, consisting of 1.25 M CuSO_4_, 0.61 M H_2_SO_4_, and CuCl_2_ 50 ppm. For the second phase an acidic solution at 60 °C of 1.25 M CuSO_4_, CuCl_2_ 50 ppm, and 0.33 g/L of high-purity GnPs was used instead. The baths were kept in agitation using a magnetic agitator located within the electrolytic cell. The agitation was set at 3 rpm. The electrodeposition process was carried out with a direct current of *I* = 3.33 A·dm^−2^. The MMCC without the third phase was mechanically removed from the aluminum substrate and observed by SEM (Zeiss LEO-Supra, Rome, Italy) and by Raman spectroscopy (Horiba LabRAM HR Evolution, Haifa, Israel). As presented in Figure 1, particles have a rigid form with an average lateral size of 15 μm in a range between 6 μm and 30 μm, and a thickness of about 8 nm. The crystalline quality of the GnPs was evaluated by their Raman spectra. The morphology and dispersion of the nanoscale fillers were verified by scanning electron microscopy (SEM). The crystalline structure of the GnPs remained intact during composite synthesis as verified by the constant D-peak intensity in Raman scattering. Tuinstra and Koening [37] were the first to study the disorder in graphitic samples in their seminal paper. The ratio of the D-peak intensity to that of the G-peak varied inversely with La (the average inter-defect distance). Thus, the lack of significant variations in the ratio between I_D_ and I_G_ indicates that no further defects were induced in the crystal lattice after the process [21]. The ratio between the D- (1360 cm^−1^) and the G-peak (1581 cm^−1^) was determined after the electrodeposition process and compared to the as-received material. As shown in Figure 1c, the D/G intensity ratio remains constant at 0.081, indicating that the electrochemical process does not induce further defects in the crystal lattice.

### 2.2. Measurements

To analyze the influence of GnPs on the composite thermal properties, the degrees of their alignment, the inclination angle and their thermal conductivity have been determined. To perform these analyses, the last layer of copper, generally used to trap the particles inside the coating (preventing them from fleeing, due to weak bonds between carbon and copper atoms), was not realized. The quantitatively orientation of carbon nanofillers inside the composite can be efficiently evaluated by polarized Raman spectroscopy [37,38,39,40,41]. The Raman’s full range spectrum used to characterize the GnPs was between 1200 cm^−1^ and 3000 cm^−1^. In this way we could analyze all the main Raman peaks for the graphite (D, G, and 2D) and perform our characterizations. Raman spectroscopy was carried out on the MMCCs without outer copper coating using a monochromatic laser wavelength of 532 nm. The maximum power on the sample was set to 1.3 mW to prevent the damaging of the sample. A series of analysis was performed to optimize the laser power. All the Raman analysis were completed with samples having only one layer of copper and one layer of copper/GnPs. Thus, the nanoparticles were directly observable by Raman spectroscopy. The angle between the polarization direction and the sample was rotated with a *λ*/2 wave plate. The incoming light, as well as the backscattered light, was focused by a 50× objective, with a grating of 1800 gr·mm^−1^ and a hole of 200. The polarization of the incoming and scattered light was chosen parallel to each other. 

To evaluate the heat conduction coefficient of a single GnP we used the Raman spectroscopy to correlate the phonon frequency shift with variations of laser power intensity and temperature [7]. The sample temperature was controlled by a cold-hot cell operated using a liquid nitrogen source. All measurements were carried out at low and constant laser excitation power to avoid overheating and complete burning of the nanoplatelets. The power on top of the cold-hot cell window was below 1.3 mW and, therefore, much smaller on the sample surface. The power density on the cold-hot cell window was measured using a Nova II OPHIR laser power meter (Haifa, Israel). The estimated accuracy of the cell temperature control was ±0.1 °C. The amount of the thermal power $\dot{Q}$ absorbed by a single graphite nanoplatelet was evaluated through the calibration procedure with HOPG, considering the reduced number of layers, absorption coefficient, scattering cross-section, and a calibration factor [42]. When the Raman laser beam is focused on the calibration HOPG sample, the measured power is ${\dot{Q}}_{D}\approx I_{0}A$, where A is the illuminated area and $I_{0}$ is the laser intensity on the surface. The scattered intensity from the HOPG sample can be obtained by summation over all n layers:

(1) $$\begin{array}{c} ∆I_{HOPG}=NI_{0}\sigma_{HOPG}\sum_{n=1}^{\infty }\exp(-2\alpha_{HOPG}a_{HOPG}n)\\ \;\approx NI_{0}\sigma_{HOPG}{\left(\exp\left(2\alpha_{HOPG}a_{HOPG}\right)-1\right)}^{-1} \end{array}$$

where $\sigma_{HOPG}$ and $\alpha_{HOPG}$ are the scattering cross-section and absorption coefficient for HOPG with the monolayer of thickness $a_{HOPG}$. The latter can be reduced to:

(2) $$∆I_{HOPG}\approx 1/2\left(N/A\right)\left(\sigma_{HOPG}/\alpha_{HOPG}a_{HOPG}\right){\dot{Q}}_{D}$$

where *N*/*A* is the surface number density of the scattering atoms. Considering a single GnP, the power absorbed is given by:

(3) $$\dot{Q}\approx I_{0}A\;exp\left(-\alpha_{GnP}a_{GnP}n\right)\approx I_{0}A$$

where $\alpha_{GnP},\;a_{GnP}$ are, respectively, the absorption coefficient and monolayer thickness of a single nanoplatelet. Thus, the Raman intensity can be related to the absorbed power as:

(4) $$∆I_{GnP}\approx 1/2\;\left(N/A\right)\;(\sigma_{GnP}/\alpha_{GnP}a_{GnP}){\dot{Q}}_{D}$$

where $\sigma_{GnP}$ is the scattering cross-section of the single nanoplatelet. Once defined, the ratio of the integrated intensities is $\varsigma =∆I_{GnP}/∆I_{HOPG}$, and the power absorbed in a graphite nanoparticle through the power measured by the detector can be expressed as:

(5) $$\dot{Q_{GnP}}=\varsigma (\sigma_{HOPG}\alpha_{GnP}a_{GnP}/\sigma_{GnP}\alpha_{HOPG}a_{HOPG}){\dot{Q}}_{D}$$

The term in the brackets is very close to unity because it consists of the ratios of the in-plane microscopic material parameters for essentially the same material. The value of ς has been determined experimentally and it completes the calibration. For several examined samples, we found ς to be in the range between 0.97 and 0.99. The value obtained mainly depended on the fact that the thickness of the GnPs used is large enough to let the nanoparticles show the same response of the material that generates them. The distribution of power between the GnPs and the copper matrix may depend on the conditions of the experiment and was checked for each experimental run.

The light flash method was used to measure thermal diffusivity (*α*) with an IPG-DRL200 laser. The power used was 20 W for a time of 0.05 s. The through-plane diffusivity was measured on the rear face of the same sample, according the Parker’s law [43], using a high-precision thermo-camera (Flir-A655SC) equipped with a 24.5 mm lens. The thermal conductivity *k* was calculated from:

(6) $$k\;=\;\alpha \rho c_{p}$$

with $c_{p}$ being the specific heat obtained by rule of mixture and ρ bulk density of the sample.

## 3. Results and Discussion

### 3.1. GnPs Alignment and Inclination

**Figure 2:**
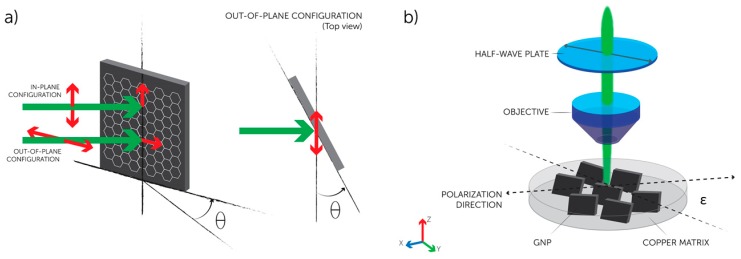
Schematic diagram of the polarization configurations and set up in our polarized Raman spectroscopy. (**a**) The inset shows the two configurations for an oblique laser incidence on a GnP plane (θ≠0) depending on the polarization direction of the incident light; and (**b**) the measurement setup for polarized Raman spectroscopy in ‘VV’ configuration.

All GnPs spectra were excited with visible (532 nm) laser light and collected in the backscattering configuration. To study the disposition of the nanofillers, it was used a polarized laser beam in ‘VV’ configuration, where the polarization direction of the incident light is parallel to the scattered light. For a single GnP, Raman scattering is allowed for an in-plane polarization, but forbidden in the perpendicular polarization direction [44]. This means that the G-peak normalized intensity will have a maximum when the polarization is parallel to the crystal lattice and a minimum when the polarization is perpendicular to it [45,46]. The intensity of the Raman active modes can be calculated using the selection rules for light scattering in crystals [44,47]. During the evaluation of a carbon crystal alignment by polarized laser beam, we can have an in-plane configuration or an out-of-plane configuration. As shown in Figure 2a, when the laser incidence is not normal, but oblique with the crystal lattice, the electric field vector of the incident light can be fully contained into a graphene plane (in-plane configuration), or it can make an angle *θ* (out-of-plane configuration), depending on the polarization direction of the incident light [45]. Considering *ε* as the specimen rotation angle with respect to the polarization direction, for perfectly aligned GnPs (i.e.,$\;\gamma_{1}$ = $\gamma_{2}$ = 0, respectively, the rotation angles of the *x*- and *z*-axis) the G-peak intensity has a maximum for the in-plane polarization (*ε* = 0, *π*, and 2*π*), while it is zero for *ε* = *π*/2, 3*π*/2. In our Raman experimental set-up presented in Figure 2b, the in-plane GnPs direction inside the matrix is oriented within the *x*-*z*-plane whereas the polarization is varied in the *x*-*y*-plane. 

**Figure 3:**
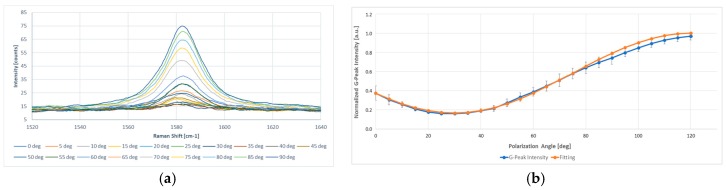
Quantitative determination of the spatial orientation of GnPs by polarized Raman spectroscopy. All Raman spectra were reduced at the same level in order to avoid any shifting and variations of the peaks due to excessive brightness of the copper surface, making comparison easier. (**a**) Intensity attenuation of the G-peak in ‘VV’ polarization configuration for a single graphite nanoplatelet; and (**b**) the comparison between actual data (blue line) and cos^2^*θ*-dependence fitting curve, with C_1_ = 0.833 and C_2_ = 0.167. The spectra are excited at 532 nm and recorded at room temperature in the ‘VV’ polarization configuration.

The normalized Raman intensity of the G-peak measured in the out-of-plane configuration, with respect to that in the in-plane configuration, was analyzed as a function of the angle *θ*. In the ‘VV’ configuration, the polarized Raman scattering on a single isolated GnP exhibits approximately cos^2^*θ*-dependence in which *θ* is the angle between the GnP basal plane and the polarization direction of the incident light. Thus, the G-peak normalized Raman intensity measured in out-of-plane configuration with respect to that measured in in-plane configuration $\frac{I_{o}\left(\theta \right)}{I_{i}\left(\theta \right)}\;\left(G\right)$ were supposed to exhibit a C_1_cos^2^*θ* + C_2_ trend, where C_1_ and C_2_ are constants such that C_1_ + C_2_ = 1 [48,49]. A similar relationship was used by Gupta et al. [50] for the intensity variation of the D-band with laser polarization angle relative to the edge of a graphene flake to consider non-uniformity of the edge. Fixed the polarization configuration, spectra were obtained with different rotations of polarization angle in steps of 5°, with the laser beam parallel to the z-axis. Experimental data of the GnPs’ inclination inside the coating, as shown in Figure 3, agreed quite well with cos^2^*θ*-dependence (orange line) as expected. 

For a single graphite nanoplatelet with a certain orientation in the matrix the inclination angle can then be calculated, considering:

(7) $$\theta \propto arccos{\sqrt{\frac{I_{min}}{I_{Max}}}}_{{TOT}_{G-Peak}}$$

**Figure 4:**
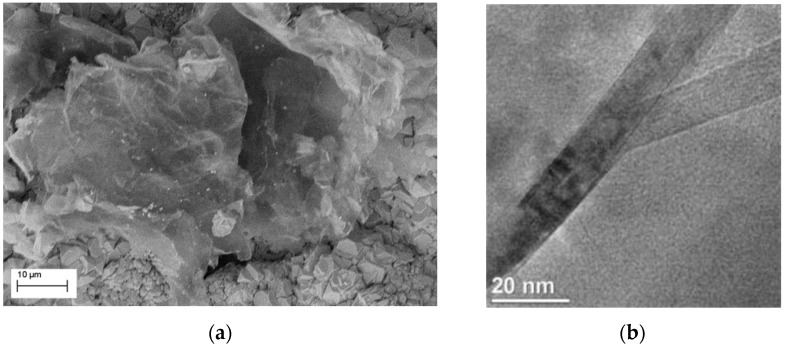
(**a**) SEM image of the disposition of a single GnP on the copper layer after the second electrodeposition phase; and (**b**) a HRTEM micrograph of a single GnP cross-plane section.

An average inclination of 66.3° ± 4.2° has been obtained for the GnPs inside the copper matrix. To determine the average statistical alignment of GnPs many different spectra were obtained rotating the polarization angle in steps of 15°, using a laser beam parallel to the *z*-direction. Since the nanoparticles have an average inclination of almost 66° in the out-of-plane configuration, the normalized intensity of the G-peak (at different polarization angles) can’t be uniform in the *x*-*y*-plane. This allows a maximum only for a polarization angle parallel to the GnP in-plane direction ($\varepsilon_{I_{G-Max}}$). Thus, a perfectly-aligned GnP ($\gamma_{1}$ = $\gamma_{2}$ = 0) has a maximum G-peak intensity only for equal polarization angle pairs. Once identified, a preferential orientation of the particles, and given to this the arbitrary value of Ε = 0° the GnPs alignment within the copper matrix can be easily determined. For each GnP analyzed, we evaluated the deviation of $\varepsilon_{I_{G-Max}}$ from the main direction individuated by polarized Raman spectroscopy (Ε). A standard deviation of ±4.16° of the $\varepsilon_{I_{G-Max}}$ from the in-plane preferred direction has been measured. Intensity variation due to crystal lattice defects and rough surfaces have also been considered. The inclination and preferred orientation direction of the GnPs within the copper matrix observed by SEM (Figure 4) and polarized Raman spectroscopy clearly influences the anisotropic thermal properties of the composite. Subsequently, the thermal conductivity of the nanoparticles ($k_{GnP}$) was measured. 

### 3.2. GnPs’ Thermal Conductivity

In carbon materials heat conduction is usually dominated by acoustic phonons, which are ion-core vibrations in a crystal lattice. This behavior is explained by the strong covalent sp^2^ bonding resulting in an efficient heat transfer by lattice vibrations [13,15,16]. For this reason, the heat conduction coefficient can be effectively determined by Raman spectroscopy correlating the phonon frequency shift with variations of laser power intensity and temperature [7]. For reasonable laser power levels, the change of phonon frequency with temperature is a manifestation of anharmonic terms in the lattice potential energy, determined by the anharmonic potential constants, the phonon occupation number, and the thermal expansion of the crystal [51]. Since the single GnP has a shorter crystal planar domain size where exist defects, it will absorb laser energy and expand easily because of its lower thermal conductivity. Consequently, the single nanoparticle shows a strong temperature dependence, also thanks to the extremely small cross-section area of the heat conduction channel due to a thickness of *h* = 8 ± 0.1 nm. The heat conduction through a surface with the cross-sectional area *S* can be evaluated from the following equation:

(8) $$\delta Q/\delta t=-k_{GnP}\oint^{\text{​}}\nabla T\;\cdot dS$$

where Q is the total heat transferred over the time *t* and *T* is the absolute temperature. For a single GnP, with planar dimensions comparable to the laser spot size light, the heat propagation through the nano-platelet can be approximated to a plane-wave heat front. In this case, the heat generated by the laser within the thin GnP can escape through a cross-sectional area S=W×h, where W  is the average particle width. Considering a uniform planar heat flow, the thermal conductivity equation can be determined by the equation:

(9) $$k_{GnP}=L/2S\cdot ∆\dot{Q}/∆T$$

where L is the distance from the middle of the GnP plane to the edge of the particle, and ∆T is the local temperature rise due to the changing heating power $∆\dot{Q}$. Since the excitation power levels are relatively low, the G-peak position linearly depends on the sample temperature by the equation:

(10) $$\omega =\omega_{0}+\chi_{G}T$$

where $\omega_{0}$ is the frequency of the G-peak when temperature *T* is extrapolated to 0 K and $\chi_{G}$ is the first-order temperature coefficient, which defines the slope of the dependence.

**Figure 5:**
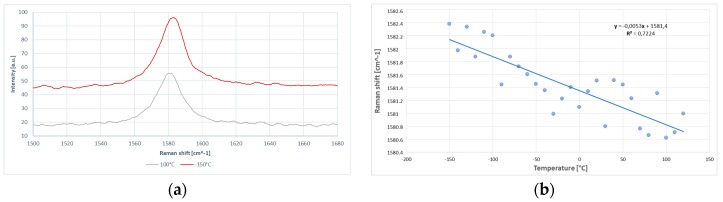
Temperature dependence of the G-peak frequency for GnPs. (**a**) The insets show the redshift of the G-peak region of the Raman spectrum from a single GnP recorded at two different temperatures; and (**b**) the measured data were used to extract the temperature coefficient for G-peak.

The final expression for the thermal conductivity in the planar heat wave case can be written as:

(11) $$k_{GnP}=\left(L/2hW\right)\cdot \chi_{G}\cdot \left(\delta \dot{Q}/\delta \omega \right)$$

where δω is a small shift in the G-peak position due to the variation $\delta \dot{Q}$ in the heating power on the sample surface. An average value of 4.48 ± 0.12 has been extracted for the ratio L/W by SEM and HRTEM morphology analysis. Several measurements varying the sample temperature have been carried out to evaluate the temperature dependence of the G-peak position. The sample temperature was controlled by a cold-hot cell operated using a liquid nitrogen source. The investigation was carried out at low and constant laser excitation power, completely avoiding the overheating and burn of the nanoplatelets. As shown in Figure 5, for a nano-graphite platelet the increasing temperature leads to the red shift of the G peak. The temperature range used to evaluate the G-peak shift was from *T* = −150 °C to *T* = 120 °C. The general trend can be clearly estimated over the examined temperature range. Some data dispersion for the G-peak shift can be mainly attributed to: drifts of the laser spot on the GnP surface due to thermal expansion of the metal matrix during the sample temperature change, high sensitivity of the G-peak to the number of graphene layers, and to the presence of defects [52]. The measurements were repeated several times on different GnPs to verify the reproducibility.

**Figure 6:**
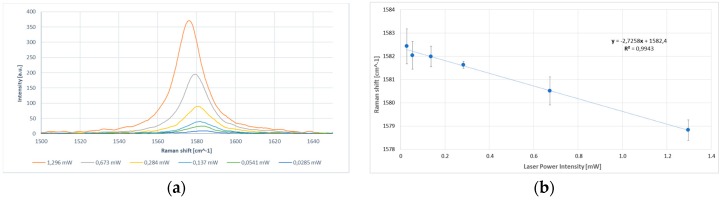
Phonons dependency with different laser power intensities. (**a**) The G peak region of the Raman spectrum from a single GnP recorded at six excitation power levels; and (**b**) the shift in G-peak spectral position due to change in total dissipated power. The spectra are excited at 532 nm and recorded at room temperature in the backscattering configuration.

The value obtained for the GnP temperature coefficient was $\chi_{G}=-\left(5.3\pm 0.28\right)\times 10^{3}$ cm^−1^·K^−1^ while the extrapolated value of $\omega_{0}$ corresponded to a frequency of 1581.4 cm^−1^. The laser low excitation power, together with the independent temperature external control, have allowed a much higher accuracy in measurements of the temperature coefficient. After determining the G-peak frequency shift due to temperature variations, the excitation power dependence of the Raman G peak was measured. The excitation power at the GnP sample location was determined by a power meter. To preserve the accuracy of our method, we measured the power at the sample position and not at the laser output to avoid any losses in the spectrometer. The power measured by the detector can be split into two terms: ${\dot{Q}}_{D}=\dot{Q_{GnP}}+\dot{Q_{Cu}}$, where $\dot{Q_{GnP}}$ is the power dissipated within the nanoparticle while $\dot{Q_{Cu}}$ is the power lost in the matrix. For cuprous oxide, there are twelve optical phonon branches theoretically expected in the Raman spectrum, and three of them are Raman active. In measured Raman spectra from copper thin films, three peaks are usually observed. They are identified as the first order phonon scattering (298, 346, and 632 cm^−1^), and assigned to A_g_ and 2B_g_ CuO phonon peaks [53]. Since the dimensions of the laser spot and the single GnP were of the same order of magnitude, we monitored the spectral region between 200 cm^−1^ and 700 cm^−1^ at different power levels to confirm that the Cu_2_O and CuO layers close to the nanoparticle were not strongly heated during the measurement. No one shift was appreciated. Therefore, the power absorbed by the single GnP was directly dependent by the one measured with the power meter. The power absorption of the single nanoplatelet can be extracted by the power absorption of the bulk HOPG considering the reduced number of layers, absorption coefficient, and scattering cross-section, and a calibration factor [42] (see Measurements). As clearly shown in Figure 6, the increase in the laser power excitation led to the increase in the intensity count and redshift of the G mode peak. 

The average value of $\delta \omega /\delta \dot{Q_{GnP}}\approx -2.73\;$ cm^−1^·mW^−1^ was finally obtained. Substituting the values extracted for the temperature coefficient $\left(\chi_{G}\right)$ and the excitation power dependence of the Raman G peak $(\delta \omega /\delta \dot{Q_{GnP})}$ in Equation (11), we obtain the average thermal conductivity value of $k_{GnP}=1244\pm 86\;$ W·m^−1^·K^−1^ for the entire set of GnPs analyzed. The thermal conductivity value obtained is in line with those available in literature for high purity graphite nano-crystals having a number of layer *n* > 8 [13]. 

### 3.3. MMCC Thermal Conductivity

Subsequently, the measurements of the thermal diffusivity (α) were carried out by the laser flash technique. For all the MMCC’s samples the diffusivity was measured in the through-plane direction, to evaluate the enhancement given by the nanoplatelets’ disposition. As a reference, the thermal diffusivity of a coating made of pure copper ($\alpha_{Cu}\approx 122\;$ mm^2^·s^−1^) was evaluated. The average value obtained for the MMCC was $\alpha_{GnP}\approx 192\;$ mm^2^·s^−1^, which is surprising for the GnP volume fraction (f)  approximately of 27%. The bulk density of the composite materials was determined by Archimedes’ principle, and is used to calculate the thermal conductivities (see Measurements) that consequently follow the same trend as the thermal diffusivities of the composites. The through-plane heat thermal conduction (HTC) obtained for the MMCC was of 640 ± 20 W·m^−1^·K^−1^, while the one obtained for the pure copper coating was of 400 ± 21 W·m^−1^·K^−1^ Despite other methods where adding highly-conductive carbon nanomaterials the thermal conduction decreased, the use of an electrochemically-controlled method has allowed an improvement of k up to 57% compared to pure copper. 

### 3.4. Effective Medium Approximation Model

**Figure 7:**
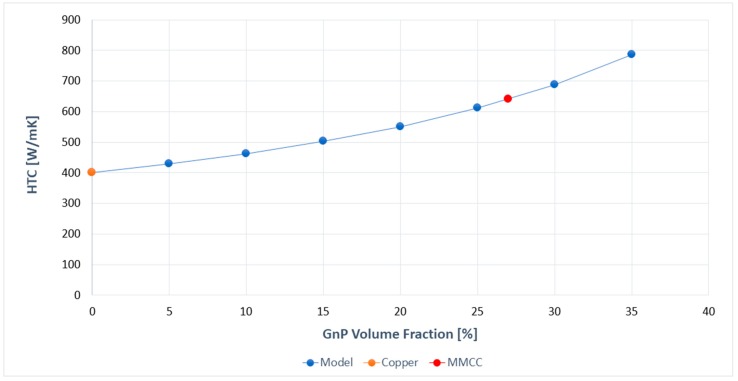
Calculated in-plane thermal conductivity enhancement for GnP-MMCC by the EMA model depending on the filler volume fraction in comparison with the experimental data obtained by the flash method.

To understand the mechanism behind the thermal conductivity enhancement, we used the model developed by Nan et al. [54] within the effective medium approximation. It describes the effect of geometry, concentration, thermal conductivity, and orientation of the filling material, as well as the thermal interface resistance between the matrix and filler on the heat conduction improvement of the composite. Considering a single GnP as an ellipsoidal oblate inclusion in the metal matrix, the effective thermal conductivity of the composite with equi-sized particles, with respect to its symmetry axes, is given by:

(12) $$\begin{array}{l} k_{11}=k_{22}\\ =k_{m}\frac{2+f\left[\beta_{11}\left(1-L_{11}\right)\left(1+{\langle \cos}^{2}\theta \rangle \right)+\beta_{33}\left(1-L_{33}\right)\left(1-{\langle \cos}^{2}\theta \rangle \right)\right]}{2-f\left[\beta_{11}L_{11}\left(1+{\langle \cos}^{2}\theta \rangle \right)+\beta_{33}L_{33}\left(1-{\langle \cos}^{2}\theta \rangle \right)\right]} \end{array}$$

and:

(13) $$k_{33}=k_{m}\frac{1+f\left[\beta_{11}\left(1-L_{11}\right)\left(1-{\langle \cos}^{2}\theta \rangle \right)+\beta_{33}\left(1-L_{33}\right){\langle \cos}^{2}\theta \rangle \right]}{1-f\left[\beta_{11}L_{11}\left(1-{\langle \cos}^{2}\theta \rangle \right)+\beta_{33}L_{33}{\langle \cos}^{2}\theta \rangle \right]}$$

with:

(14) $$\beta_{ii}=\frac{k_{ii}-k_{m}}{k_{m}+L_{ii}\left(k_{ii}-k_{m}\right)}$$

and:

(15) $${\langle \cos}^{2}\theta \rangle =\frac{\int^{\text{​}}\rho \left(\theta \right)\cos^{2}\theta \sin\theta d\theta }{\int^{\text{​}}\rho \left(\theta \right)\sin\theta d\theta }$$

where θ is the angle between the materials axis Z and the local particle symmetric axis $Z^{\prime }$, ρ(θ) is a distribution function describing ellipsoidal particle orientation, $k_{ii}$ and $k_{m}$ are, respectively, the thermal conduction of the particle and of the matrix, and $L_{ii}$ are geometrical factors dependent upon the particle shape. To calculate $k_{11}$ and $k_{33}$ we used the values measured by Raman spectroscopy and the laser flash technique, widely explained above, with $k_{GnP_{11}}=k_{GnP_{22}}=1244\;$ W·m^−1^·K^−1^, $k_{GnP_{33}}=15\;$ W·m^−1^·K^−1^, and $k_{m}=400\;$ W·m^−1^·K^−1^. In order to match the experimental data, the Kapitza resistance has been neglected. This indicates that the Kapitza resistance is not a limiting factor for a high thermal conductivity of a copper-GnP composite, since the total thermal resistance is not dominated by the Kapitza resistance. The EMA calculations are in excellent agreement with the experimental observations (Figure 7). 

This shows that the alignment and inclination of the fillers is the major factor for the high through-plane thermal conductivity obtained. Additional improvements can be done increasing the inclination of the nanoparticles, together with a further increase of the lateral size of GnP and the inclusion of fillers with higher intrinsic thermal properties, like graphene sheets (GS) with $k_{GS}\approx 2000–5000\;$ W·m^−1^·K^−1^.

## 4. Conclusions

We characterized, for the first time, the thermal conductivity of the composite coating starting from the nanofillers. We proposed a simple approach to evaluate the inclination, the alignment and anisotropic phonon thermal conductivity of the nanoparticles inside the copper matrix by Raman spectroscopy. Moreover, the thermal conductivity of the composite coating has been measured by the flash method. Through the EMA-model we correlated the nanoparticle properties to the metal matrix, and the global experimental results obtained for the heat conduction of the coating. Our experimental results, together with the EMA modulations, suggest that a further increase of the GnP lateral size, with an improvement of the nanoparticle’s inclination, may result in a higher thermal conductivity, far superior than the one of pure copper. The MMCC developed, with very high thermal conductivity, have great potential for high power density heat spreading applications.
